# Supplementary material for: Mechanisms governing the pioneering and redistribution capabilities of the non-classical pioneer PU.1
Source: Nat Commun. 2020 Jan 21;11:402. doi: 10.1038/s41467-019-13960-2 (PMC6972792; doi:10.1038/s41467-019-13960-2)
Supplement: Supplementary file 7 — Source data [file 41467_2019_13960_MOESM7_ESM.zip › Source_Data/FigureS2/FigureS2B_MotifScanOutput/KmeansCluster6/homerResults/motif7.similar.html]

motif7

## Information for motif7

T
A
G
C
C
G
T
A
C
T
A
G
G
C
A
T
C
A
G
T
G
A
T
C
A
G
C
T
A
G
T
C
T
C
G
A
A
G
C
T
C
T
A
G
C
T
G
A
C
A
T
G
T
C
G
A
  
Reverse Opposite:  

A
G
C
T
G
T
A
C
G
A
C
T
G
A
T
C
T
C
G
A
A
G
C
T
T
C
A
G
C
T
G
A
C
T
A
G
G
T
C
A
C
G
T
A
A
G
T
C
G
C
A
T
A
T
C
G
  

|  |  |
| --- | --- |
| p-value: | 1e-96 |
| log p-value: | -2.217e+02 |
| Information Content per bp: | 1.682 |
| Number of Target Sequences with motif | 209.0 |
| Percentage of Target Sequences with motif | 6.35% |
| Number of Background Sequences with motif | 338.3 |
| Percentage of Background Sequences with motif | 0.79% |
| Average Position of motif in Targets | 112.8 +/- 41.9bp |
| Average Position of motif in Background | 97.8 +/- 76.4bp |
| Strand Bias (log2 ratio + to - strand density) | -0.2 |
| Multiplicity (# of sites on avg that occur together) | 1.40 |
| Motif File: | file (matrix) reverse opposite |

### Similar de novo motifs found

|  |  |  |  |  |  |  |  |
| --- | --- | --- | --- | --- | --- | --- | --- |
| Rank | Match Score | Redundant Motif | P-value | log P-value | % of Targets | % of Background | Motif file |
| 1 | 0.967 | C G T A C T A G G C A T A C G T G T A C G A C T G A T C T C G A G A C T C T A G C T G A A C T G T C G A | 1e-87 | -201.820424 | 6.89% | 1.10% | motif file (matrix) |
| 2 | 0.942 | A T C G G C A T A C G T G T A C G A C T A G T C T C G A A G C T T C A G C T G A C A T G C G T A | 1e-81 | -186.873660 | 5.74% | 0.79% | motif file (matrix) |
| 3 | 0.873 | A C G T A G T C C T G A A G C T C T A G C G T A A C T G C G T A C G A T A G T C | 1e-61 | -142.725612 | 5.59% | 1.06% | motif file (matrix) |
| 4 | 0.790 | A C G T G T A C A C G T A G T C C T G A A G C T C T A G C G T A A C T G | 1e-42 | -97.915706 | 5.04% | 1.27% | motif file (matrix) |
